# Pathogenesis and contemporary diagnoses for lateral hip pain: a scoping review

**DOI:** 10.1007/s00167-020-06354-1

**Published:** 2020-12-19

**Authors:** Karadi Hari Sunil Kumar, Jaikirty Rawal, Naoki Nakano, André Sarmento, Vikas Khanduja

**Affiliations:** 1 Addenbrooke’s - Cambridge University Hospitals NHS Foundation Trust, Hills Road, Box 37, Cambridge, CB2 0QQ UK; 2 Orthopaedic Department, Centro Hospitalar Vila Nova de Gaia, Vila Nova de Gaia, Portugal

**Keywords:** Hip, Abductor tear, External snapping hip syndrome, Greater trochanteric pain syndrome

## Abstract

**Purpose:**

Recent advances in diagnostic imaging techniques and soft tissue endoscopy now allow for precise diagnosis and management of extra-articular hip pathology. The aim of this scoping review is to present an evidence-based update of the relevant literature focussing only on the pathoanatomy, clinical assessment and the diagnosis of pathology in the peritrochanteric space.

**Methods:**

A literature search was performed on PubMed to include articles which reported on the anatomy and diagnosis of greater trochanteric pain syndrome, trochanteric bursitis, gluteus medius tears and external snapping hip syndrome.

**Results:**

A total of 542 studies were identified, of which 49 articles were included for full text analysis for the scoping review. Peritrochanteric space pathology can be broadly classified into (1) greater trochanteric pain syndrome (GTPS), (2) abductor tears and (3) external snapping hip syndrome. Anatomically, gluteus medius, gluteus minimus and tensor fascia lata work in conjunction to abduct and internally rotate the hip. The anterolateral part of the gluteus medius tendon is more prone to tears due to a thin tendinous portion. Increased acetabular anteversion has also been shown to be associated with gluteal and trochanteric bursitis. In terms of clinical examination, tests which were found to be most useful for assisting in the diagnoses of lateral hip pain were the single-leg stance, resisted external derotation of the hip, hip lag sign and the Trendelenburg’s test. Dynamic ultrasound along with guided injections and MRI scan do assist in differentiating the pathology and confirming the diagnosis in patients presenting with lateral hip pain. Finally, the assessment of baseline psychological impairment is essential in this group of patients to ensure outcomes are optimised.

**Conclusion:**

Lateral hip pain used to be a poorly defined entity, but advances in imaging and interest in sports medicine have led to a better understanding of the pathology, presentation and management of this cohort of patients. A thorough appreciation of the anatomy of the abductor musculature, specific clinical signs and imaging findings will lead to an appropriate diagnosis being made and management plan instituted.

**Level of evidence:**

IV.

## Introduction

Intra-articular hip arthroscopy has expanded rapidly over the last two decades in the management of pathology with an aim not only to treat, but also to preserve the normal architecture of the joint [32]. Furthermore, continual advances in technology and surgical technique coupled with a greater understanding of extra-articular pathologies have contributed to the development of extra-articular hip endoscopy [41]. Further improvements in diagnostic interpretation of radiographs, dynamic ultrasound, MRI and CT imaging have helped delineate the causes for anterior, lateral and posterior hip pain, thereby helping identify specific treatable pathology around the hip [10, 11, 14, 28].

Lateral hip pain is one of the common symptoms with which patients present to the hip clinic. Accurate identification of the pathology is vital to ensure appropriate treatment. Lateral compartment of the hip is defined as the space between the greater trochanter and the tensor fascia lata [2] and arthroscopy of this compartment is increasingly performed in addition to traditional hip arthroscopy [16, 17]. This extra-articular procedure provides a valuable tool for assessing and addressing pathology in the periarticular region of the hip.

The aim of this scoping review is to present an evidence-based update of the regional anatomy and the relevant literature focusing only on the pathoanatomy, clinical assessment and the diagnosis of pathology in the peritrochanteric space.

## Materials and methods

### Scoping review: Identification of studies

A literature search was performed on PubMed to include articles from inception to 30th of June 2020 using the keywords in various combinations as shown in Table 1. Studies reporting on the anatomy and diagnosis of greater trochanteric pain syndrome, trochanteric bursitis, gluteus medius tears and external snapping hip syndrome (ESHS) were included for analysis. Studies which focused on only on the anatomy and diagnosis of lateral hip pain were included as well. Narrative reviews, case reports and studies focussing on treatment or outcomes were excluded from the analysis.

**Table 1:** Search terms used for scoping review

Keywords combination	Results
(((diagnosis and (greater trochanteric pain syndrome)) OR (diagnosis and (external snapping hip syndrome)) OR (diagnosis and (gluteus medius tears)) OR (diagnosis and (trochanteric bursitis)))	388
(((anatomy and (greater trochanteric pain syndrome)) OR (anatomy and (external snapping hip syndrome)) OR (anatomy and (gluteus medius tears)) OR (anatomy and (trochanteric bursitis)))	133

## Results

**Fig. 1:**
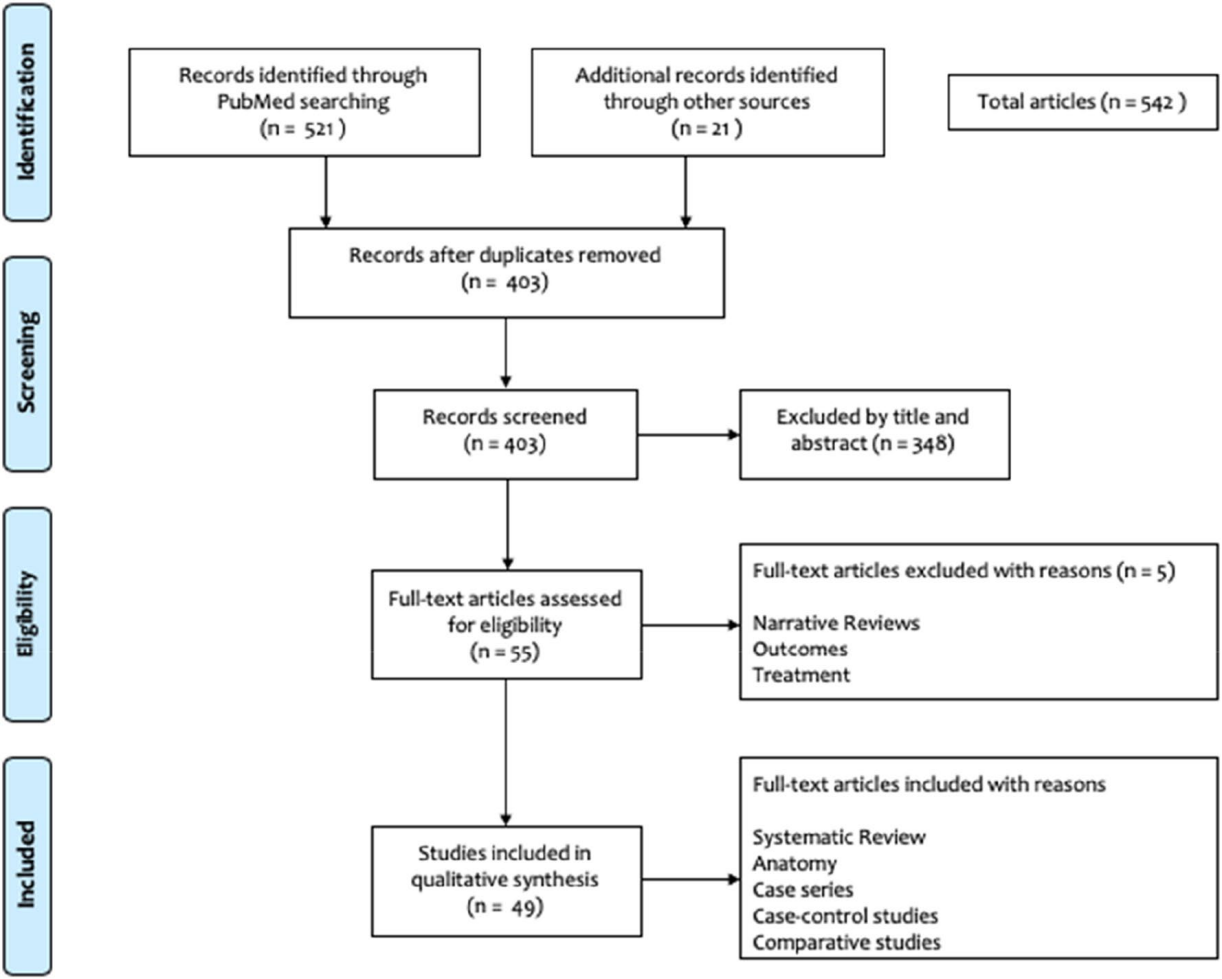
PRISMA flowchart

A total of 542 studies were identified from the initial search. Following a thorough screening, 49 articles were finally included for full text analysis of the scoping review. The PRISMA flowchart for the scoping review is shown in Fig. 1.

### Anatomy of the hip abductor complex

**Fig. 2:**
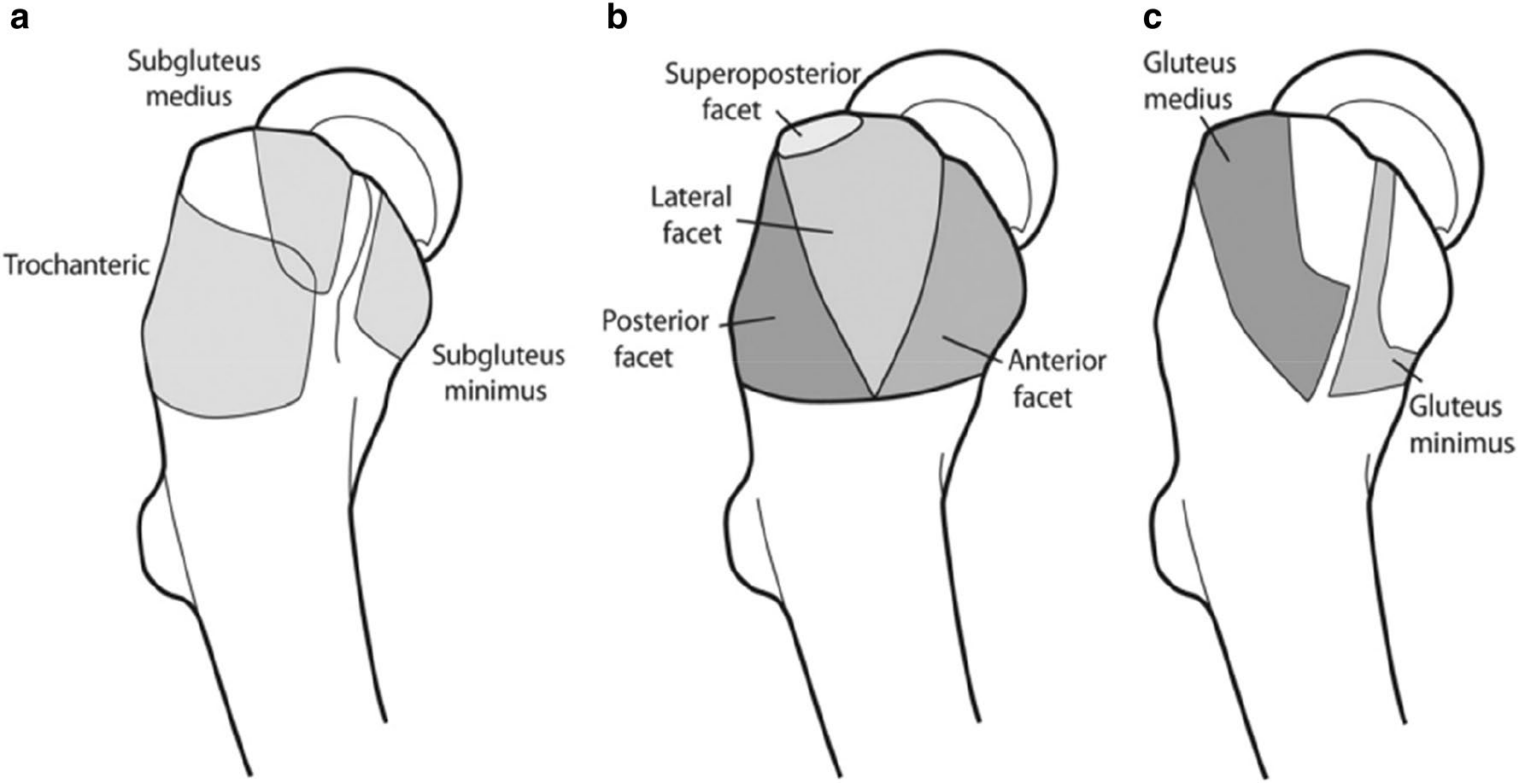
Anatomy of the greater trochanter with tendinous insertion sites. **a** Three main bursae around the GT, **b** facets of the GT and **c** tendinous insertions over GT. Reprinted with permission from Arthroscopy Domb BG et al. [19]. (*GT* greater trochanter)

The peri-trochanteric space is the interval between the greater trochanter and a sleeve of muscles—gluteus maximus, tensor fascia lata and iliotibial band [6]. The hip abductor muscles, which form the basis of the peritrochanteric space, comprise the gluteus medius, gluteus minimus, and tensor fascia lata [21]. The greater trochanter (GT), which is the insertion site of the abductor muscle complex, has four facets (Fig. 2b): anterior, lateral, posterior and superior-posterior facets with insertions from different tendons [45].

#### Gluteus medius

Gluteus medius is the largest and perhaps the most complex of the three hip abductors. This muscle has three areas of origin: the gluteal fossa, the gluteal aponeurosis, and the posterior inferior edge of the lip of the iliac crest. There are also three points of insertion of the gluteus medius tendon (Fig. 2c). The tendinous portion of the aponeurosis inserts onto the superolateral facet of the greater trochanter, whilst the remainder inserts along an anteroinferior oblique line running on the lateral facet [50]. In Flack’s cadaveric investigation, the majority of muscle fascicles inserted onto the deep portion with a more proximal insertion on the greater trochanter than the superficial part of the tendon [22]. A small cadaveric series found that the tendon length was significantly longer in males, but the gross muscle volume was not significantly different on a gender basis [21]. Innervation of gluteus medius is from branches of the superior gluteal nerve, which passes between the gluteus minimus and medius, pierces and then branches in the gluteus medius, and finally enters the tensor fasciae lata (TFL) in its mid length [22]. Gluteus medius abducts and internally rotates the hip joint, and stabilises the pelvis during locomotion, thereby preventing a pelvic drop. Tsutsumi et al. evaluated 25 hips in 15 Japanese cadavers and found that the anterolateral part of the gluteus medius tendon was thin compared to the posterior part which may well be the reason for this area being more prone to tears [54].

#### Gluteus minimus

The origin of the gluteus minimus is largely bony from the gluteal surface of the ilium, from the anterior to the inferior gluteal line. The sciatic notch represents the posterior boundary, whilst the anterior boundary is the anterior edge of the ilium. Some fascicles arise from the fascia of the hip joint capsule, whilst some arise from the reflected head of the rectus femoris. Overall insertion is on the anterior facet of the greater trochanter (Fig. 2). However, Flack et al. did recognise that with a small number of their cadaveric specimens, gluteus minimus inserted into the piriformis. Gluteus minimus is also innervated by the superior gluteal nerve. It helps with abduction and internal rotation of the lower limb, along with gluteus medius and helps with stabilising the pelvis during locomotion.

#### Tensor fascia lata

Tensor fascia lata (TFL) is a flat tendon enveloped in the fascia lata with the proximal tendon arising from the anterior superior iliac spine (ASIS), mostly from the anterior and lateral borders. Insertion of its fascicles is onto the fascia itself, forming a condensation of fascia known as the iliotibial band. The iliotibial band (ITB) also has a point of tendinous insertion of the gluteus maximus muscle. Innervation of TFL is from branches of the superior gluteal nerve entering the TFL in its mid length [21]. The TFL, in conjunction with gluteus medius and minimus, works to abduct and internally rotate the hip and with the rectus femoris helps with hip flexion. The proximal part of the ITB helps with hip extension, hip abduction and external rotation of the hip [29].

#### Gluteal bursae

Each of the above tendon insertion is associated with its own bursa and therefore there are three bursae in this region—a subgluteus medius bursa overlying the superior part of the lateral facet, a gluteus minimus bursa over the lateral facet and a subgluteus maximus bursa between the gluteus maximus and the ITB (Fig. 2a) [45].

### Function of the hip abductors

Hip abductor function is more complex than simply stabilising the pelvis during gait. Gluteus medius function is likely to vary depending on the tasks demanded of it during the gait cycle (pelvic stabilisation) or in concentric or eccentric control of abduction and external rotation or adduction and internal rotation. The function of the abductors is tested with the Trendelenburg test [8]. Gluteus minimus acts as a flexor, abductor and an internal rotator of the hip depending on the position of the thigh [3]. Gottschalk et al. using EMG studies identified that the primary function of the gluteus minimus and the posterior fibres of the gluteus medius is stabilisation of the femoral head and the acetabulum during the gait cycle [25].

### Peritrochanteric pathology

There are several pathologies that can affect the abductor muscle complex in the peritrochanteric space which can be broadly classified into (1) greater trochanteric pain syndrome (GTPS), (2) abductor tendon (gluteus medius/minimus) tears and (3) external snapping hip syndrome (ESHS).

#### Greater trochanteric pain syndrome (GTPS)

GTPS is thought to be a degenerative condition affecting the abductor tendons and the bursae around them [20, 34]. GTPS has a female preponderance of 4:1 with the majority of the patients between the fourth and sixth decades of life [57]. A varus femoral neck and females with increased gynoid adipose tissue are at a higher risk of developing GTPS [20]. Spontaneous onset of lateral hip pain with point tenderness over the GT is suggestive of GTPS [57].

**Fig. 3:**
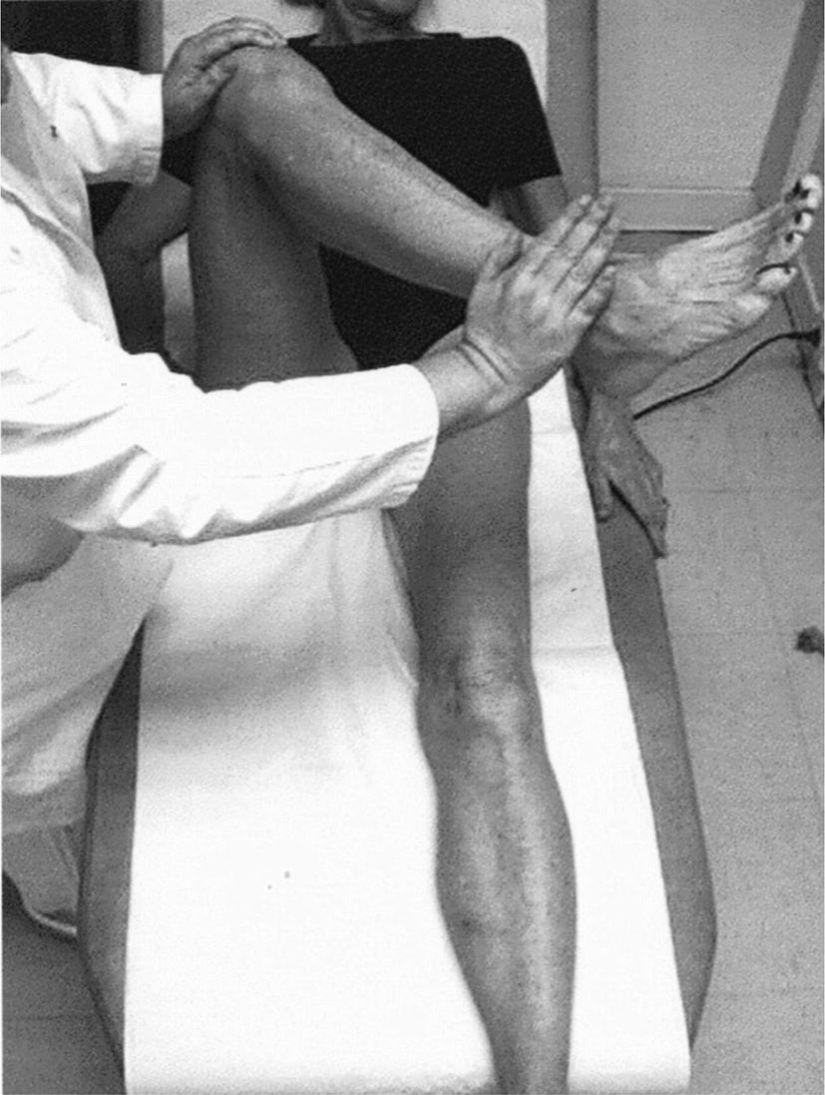
Resisted external derotation test. Reproduced from Gluteal tendinopathy in refractory greater trochanter pain syndrome: Diagnostic value of two clinical tests Lequesne et al. Arthritis Care and Research Jan 31 2008, reproduced with permission from John Wiley and sons

**Fig. 4:**
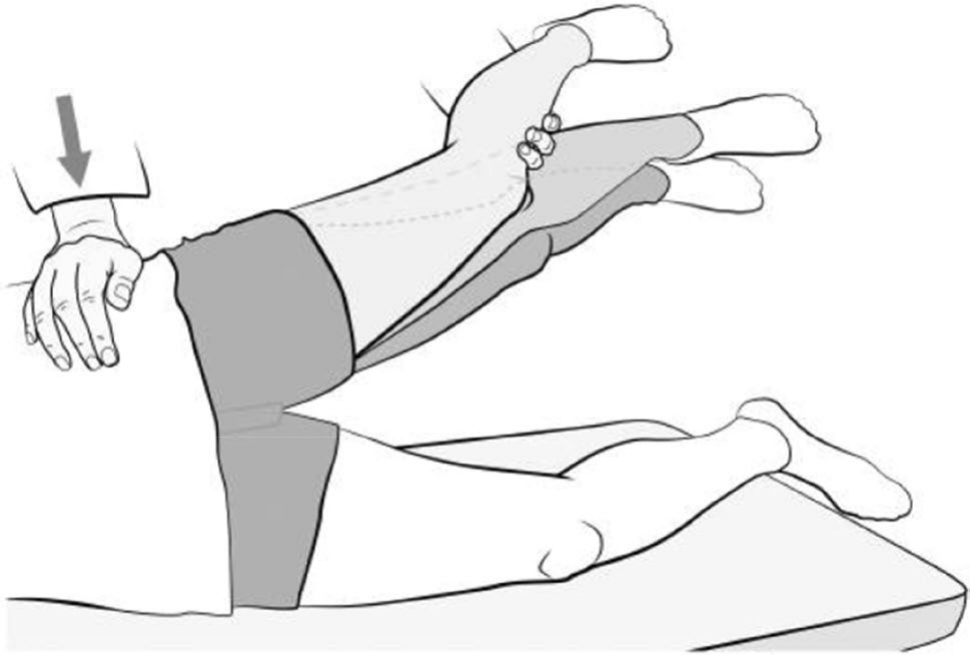
Hip lag sign. Reproduced from Kaltenborn et al. [31]. Reproduced under the terms of the Creative Commons Attribution License

Clinical tests which help in the diagnosis of GTPS are inability to complete a single-leg stance for 30 s and inability to perform a resisted external derotation of the hip in the supine position with the hip flexed to 90° (Fig. 3) and in the prone position with hip extended. The resisted external derotation test is performed with the hip flexed to 90° and the hip being placed in full internal rotation and then the patient being asked to bring the leg to a neutral position against resistance. A positive test is indicated by pain or weakness. The sensitivity and specificity for single-leg stance was 100% and 97.3% and that for resisted external derotation of the hip in supine position was 88% and 97.3%, respectively, as correlated in the MRI study by Lequesne et al. [37]. This was also supported by the study by Ganderton et al., who reported that a positive hip flexion, abduction and external rotation (FABER) test, tenderness over GT, and a positive resisted hip abduction and external derotation test supported the diagnose of GTPS [24]. Furthermore, a hip lag sign (Fig. 4), when correlated with an MRI, has been shown to be accurate in diagnosing hip abductor damage, with a sensitivity, specificity, PPV and NPV of 89%, 96%, 94% and 93%, respectively [31].

Plain radiographs are useful to rule out calcific tendinitis in the abductor tendons, arthritis in the hip joint, femoroacetabular impingement (FAI) or any other morphological abnormality and other intra-articular pathologies causing hip pain. In patients younger than 40 years, pincer-type FAI, which is defined as acetabular overcoverage with the lateral centre-edge angle ≥ 39 degrees, may be associated with an increased incidence of GTPS [48].

Ultrasound scan (US) can be useful in identifying any peri-trochanteric pathology, but is very much operator dependent. A large study of 877 ultrasounds of hips for lateral hip pain reported 20% had trochanteric bursitis, 29% had ITB thickening or tears, and nearly 50% had gluteal tendinosis [40].

MRI has been reported by several authors to help confirm the diagnosis of GTPS with the demonstration of peritrochanteric oedema on T2 sequences. Haliloglu et al. in their series of 79 patients reported that 70% had peritrochanteric oedema, out of which 95% had bilateral changes [26]. On the other hand, Blankenbaker et al. concluded that the presence of peri-trochanteric abnormalities on T2 MRI was not absolutely conclusive of GTPS, but an absence of these findings ruled out the possibility of GTPS [5]. Chi et al. demonstrated in their retrospective MRI study on 185 patients that with increasing age, gluteus medius and minimus tendinopathy progressed to atrophy and subsequent tears [13].

**Fig. 5:**
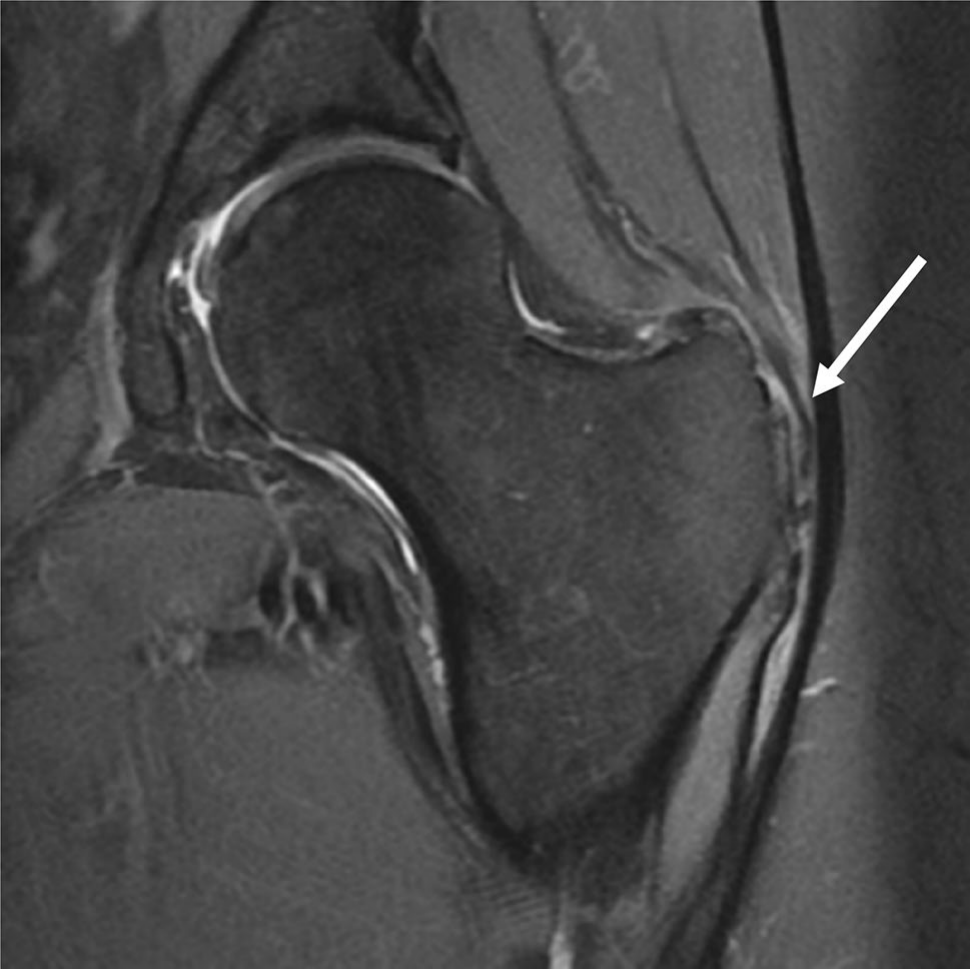
MRI T2 sequence showing hyperintensity around the greater trochanter, signifying GTPS

US and MRI have been shown to help with the diagnosis of GTPS, which has been supported by Westacott et al. in their systematic review reporting a sensitivity of 33–100% for MRI and 79–100% for US to diagnose GTPS. In addition, MRI had a high specificity of 92–100% for diagnosing GTPS (Fig. 5). However, ultrasound was shown to have a higher positive predictive value of 95–100% compared to 71–100% for MRI to diagnose GTPS. Therefore they suggested US, performed by an experienced musculoskeletal radiologist, as the investigation of choice [56].

In a large epidemiological study of 5735 lower limbs in 2954 subjects, Segal et al. reported that 60% cases were women and the prevalence rate was 11.7% for unilateral and 5.9% for bilateral GTPS [51]. In addition, they concluded that altered lower limb biomechanics may be inter-related to GTPS. Increased acetabular anteversion (AA) has been shown to be associated with gluteal tendinopathy (AA = 18.4°) and trochanteric bursitis (AA = 18.8°) compared to controls (AA = 15.4°) [44]. Increased AA may alter the biomechanics of gluteal tendons which may have a bearing on GTPS. In addition, Canetti et al. reported the association of low sacral slope with GTPS in their case control study and that patients with GTPS were likely to suffer from low back pain (LBP) (49%) [7]. They postulate that the decrease in sacral slope changed the biomechanics of the gluteal tendons by retroverting the pelvis. Careful assessment of patients with low back pain is vital to avoid overlooking the diagnosis of GTPS, as Tortolani et al. have reported approximately 20% in their series of 247 consecutive patients with LBP had GTPS [53]. Furthermore, hypermobility is seen more frequently in patients diagnosed with GTPS comprising 11% of males and 25% of females which may have an influence on treatment outcomes [49].

Patients with FAI who had a lower baseline psychological impairment were shown to have an inferior outcome compared with those who did not suffer from mental health disorders [12]. Similarly, Plinsinga et al. concluded in their article that patients with persistent GTPS had greater psychological distress, physical impairments and poor health-related quality of life, which were associated with pain and disability [46]. Furthermore, Plinsinga et al. reported that patients with GTPS had greater body mass index, lower peak muscle strength, short step length and low walking speed [47]. These factors need thorough assessment to achieve good outcomes following treatment for GTPS.

#### Abductor muscle tears

Analogus to the rotator cuff of the shoulder, tears of the gluteus medius tendon insertion (more common) or gluteus minimus tendon insertion (less common) have been described and thought to contribute to the presentation of GTPS. Tears can be partial, complete or intra-substance. Partial tears usually occur on the under-surface, comparative to the articular-sided rotator cuff partial tear. The incidence of abductor tendon atrophy increases beyond the age of 50 years which may in turn result in tears, with a mean age of gluteus medius tears being 54 years with a female preponderance [13, 39]. Bunker et al. reported a prevalence of 22% of abductor tears in patients who sustained fracture of the neck of the femur [27]. The presence of lateral hip pain, tenderness over greater trochanter, weakness of abductor function, pain reproduced on resisted hip abduction, tenderness over the gluteus medius muscle and a positive Trendelenburg test are all suggestive of abductor muscle tears [39, 57]. The diagnosis has been facilitated with increased recognition and improvements in MRI as discussed in the previous section. Access to the undersurface tear, however, is more fraught with iatrogenic injury to the tendinous insertion of the abductors.

Thorough clinical examination may indeed help diagnose abductor muscle tears. Bird et al. reported that Trendelenburg test was the most accurate test, compared with pain on resisted hip abduction and pain on resisted internal rotation (IR) of the hip, to diagnose a gluteus medius tear. The intra-observer reliability kappa scores were as follows: Trendelenburg's sign 0.676 (95% confidence interval [95% CI] 0.270, 1.08), resisted hip abduction 0.625 (95% CI 0.155, 1.09), and resisted hip internal rotation 0.027 (95% CI − 0.016, 1.10) [6]. However, they suggest an MRI scan to confirm the diagnosis, especially prior to considering surgical treatment for this condition. Furthermore, Ortiz-Declet et al. [43] showed that Trendelenburg gait had 100% specificity and 100% positive predictive value compared to resisted internal rotation test for gluteus medius tears which had a specificity and PPV of 85% each. However, resisted IR test was shown to have an approximately 90% sensitivity, 85% specificity, and 92% NPV when used in conjunction with an MRI for gluteus medius tears [43]. Chandrasekaran et al. reported that those patients with a reduced power of resisted abduction of less that grade 4/5 MRC grade and the presence of a Trendelenburg gait increased the likelihood of the need for surgical repair by a 14-fold increase [9]. Walker-Santigo et al. have described the use of resisted IR of the hip to identify gluteus medius tendon tears [55]. However, they did not report the sensitivity and specificity of this test.

MRI scans have been used more frequently, when compared to US, to diagnose abductor muscle tears. Cvitanic et al. in their study showed that MRI was accurate in diagnosing abductor tendon tears and that abductor tendon atrophy was seen with large tears. Hyperintensity superior to the greater trochanter on T2 MRI was shown to have a sensitivity of 73% and specificity of 95% for abductor tendon tears [15]. Sutter et al. in their study of 35 patients who underwent MRI of abductor tendons reported that 46% had either gluteus medius or minimus tendon tears. In addition, they reported hypertrophy of the TFL muscle in those with abductor tendon tears in comparison to the contralateral healthy side [52]. Furthermore, Chi et al. reported that with advancing age there is an increase in abductor muscle atrophy with progression of tendinosis to low- and high-grade tears [13]. A comparative study of US versus MRI, between patients with abductor tendon pathology and matched controls, reported that both US and MRI were able to reasonably identify abnormalities. However, it should be borne in mind that both modalities had limitations in differentiating tendinosis and partial abductor tears [18].

#### External snapping of the hip

External snapping hip syndrome (ESHS) is a condition arising from ITB thickening in its posterior part or anterior tendon fibres of the gluteus maximus near its insertion [29]. Approximately, 5–10% of the general population complain of ESHS with women being more affected that men [23, 38]. Ballet dancers had a high incidence of ESHS (91%) with 80% having bilateral involvement [38]. Repeated extreme range of movement could predispose to ESHS. Hypermobility defined as a Beighton's score of ≥ 6 increased the risk of developing ESHS due to repeated irritation of the ITB over the GT [4]. In a normal hip during flexion, the ITB passes from posterior to anterior over the greater trochanter (GT) and is brought back posteriorly on extension. However, with thickening of the ITB, this movement becomes abnormal with the tendinous glide becoming defective and characterised by an audible and often visible snapping when passing from posterior to anterior or vice versa during the flexion–extension cycle. In hip extension, the thickened portion of the ITB or gluteus maximus remains posterior to the greater trochanter. On flexion, the TFL passes past the greater trochanter to lie anteriorly and is brought back posteriorly with extension. Associated with the change in ITB pattern of movement, ITB bursal hypertrophy and irritation of the vastus lateralis tendinous origin distally on the greater trochanter may exist [30]. In addition, adduction or internal rotation of the hip brings about a similar movement of the ITB resulting in a snapping sensation. ESHS is associated with repetitive physical activity or chronic use involving extremes of range of movement of the hip [38]. Furthermore, some anatomical features such as a varus femoral neck angle, increased distance between greater trochanters or prominent greater trochanters may predispose to ESHS [1, 36].

Most commonly, the patients with ITB tightness complain of lateral hip pain during activities of hip movement such as walking, jogging and cycling. The snapping sensation may or may not be present. In addition, lateral knee pain can be reported which is aggravated with repetitive activity [57]. Some patients may complain of a feeling of subluxation or dislocation of the hip, which occurs during snapping [38].

The snapping sensation can be elicited in the clinic by asking the patient to move the hip from flexion, abduction and external rotation (FABER) into extension, adduction and internal rotation or return to neutral rotation [42]. Passive movement of the extended, adducted and internal position to FABER may also elicit the snapping sensation. In addition, the Ober's test can be performed to evaluate ITB tightness with the patient in the lateral decubitus, with the affected side up. The hip is actively flexed followed by passive extension and abduction resulting in a snap suggestive of ESHS. Furthermore, the hula-hoop test, in which the patient stands up, adducts and circumducts the affected hip resulting in snapping over the greater trochanter is suggestive of ESHS. Kim et al. classified ESHS into three grades depending on the clinical symptoms as shown in Table 2 [33].

**Table 2:** Grades of ESHS based on the degree of snapping from patient symptoms and clinical signs— reproduced from Kim et al. [33]

Grade	Degree of snapping
I	Patient complaints of slight or occasional popping symptom on trochanteric area, but not definitely palpated on physical examination
II	Definitely palpable popping symptom on physical examination continuously, but not visually seen
III	Marked snapping symptom visually seen or occasionally accompanied by audible sound

Ultrasound examination is often useful to aid diagnosis, when snapping is not elicitable in clinic, to visualise the TFL gliding over the GT [42]. This is however operator dependent for identifying the pathology. Several authors have reported that a dynamic ultrasound is useful in making a definitive diagnosis by confirming an audible or palpable snap or pain reported by the patient on the provocative manoeuvre of the hip [10, 11, 13, 35, 44]. MRI scan can be a useful investigation to aid diagnosis when one is unable to confirm ESHS clinically. Thickening and hyperintensity of the proximal iliotibial tract or hypertrophy of the gluteus maximus tendon insertion to the TFL on the T2 sequence is suggestive of ESHS [23]. Hyperintense greater trochanteric bursa or a fluid collection visualised on T2 may also suggest ESHS [23]. In an MRI based study evaluating 55 patients with ESHS, it was classified as grade I in 25.5%, grade II in 40% and grade III 34.6%. They also found difference in ESHS pathology depending on the location of the abnormality with ITB being tensed and gluteus maximum being hypertrophied. On the whole a dynamic ultraound by an experienced operator and a MRI scan are useful for confirming the diagnosis and aiding the management plan for ESHS.

## Conclusion

Lateral hip pain used to be a poorly defined entity, but advances in imaging and interest in sports medicine have led to a better understanding of the pathology, presentation and management of this cohort of patients. A thorough appreciation of the anatomy of the abductor musculature, specific clinical signs and imaging findings will lead to an appropriate diagnosis being made and management plan instituted. Finally, it should be noted that assessment of baseline psychological impairment is essential in this group of patients to optimise outcomes.

## References

[1] Allen WC, Cope R (1995). Coxa saltans: the snapping hip revisited. J Am Acad Orthop Surg.

[2] Audenaert E, Pattyn C (2009). Balloon dissection for improved access to the peritrochanteric compartment. Arthroscopy.

[3] Beck M, Sledge JB, Gautier E, Dora CF, Ganz R (2000). The anatomy and function of the gluteus minimus muscle. J Bone Joint Surg Br.

[4] Beighton P, Horan F (1969). Orthopaedic aspects of the Ehlers-Danlos syndrome. J Bone Joint Surg Br.

[5] Blankenbaker DG, Ullrick SR, Davis KW, De Smet AA, Haaland B, Fine JP (2008). Correlation of MRI findings with clinical findings of trochanteric pain syndrome. Skeletal Radiol.

[6] Byrd JWT (2015). Disorders of the peritrochanteric and deep gluteal space: new frontiers for arthroscopy. Sports Med Arthrosc Rev.

[7] Canetti R, de Saint VB, Vieira TD, Fière V, Thaunat M (2020). Spinopelvic parameters in greater trochanteric pain syndrome: a retrospective case–control study. Skeletal Radiol.

[8] Cassidy L, Bandela S, Wooten C, Jennifer C, Tubbs RS, Loukas M (2014). Friedrich Trendelenburg: historical background and significant medical contributions. Clin Anat.

[9] Chandrasekaran S, Vemula SP, Gui C, Suarez-Ahedo C, Lodhia P, Domb BG (2015). Clinical features that predict the need for operative intervention in gluteus medius tears. Orthop J Sports Med.

[10] Chang CY, Kreher J, Torriani M (2016). Dynamic sonography of snapping hip due to gluteus maximus subluxation over greater trochanter. Skeletal Radiol.

[11] Chang K-S, Cheng Y-H, Wu C-H, Özçakar L (2015). Dynamic ultrasound imaging for the iliotibial band/snapping hip syndrome. Am J Phys Med Rehabil.

[12] Cheng AL, Schwabe M, Doering MM, Colditz GA, Prather H (2020). The effect of psychological impairment on outcomes in patients with prearthritic hip disorders: a systematic review and meta-analysis. Am J Sports Med.

[13] Chi AS, Long SS, Zoga AC, Read PJ, Deely DM, Parker L, Morrison WB (2015). Prevalence and pattern of gluteus medius and minimus tendon pathology and muscle atrophy in older individuals using MRI. Skeletal Radiol.

[14] Choi YS, Lee SM, Song BY, Paik SH, Yoon YK (2002). Dynamic sonography of external snapping hip syndrome. J Ultrasound Med.

[15] Cvitanic O, Henzie G, Skezas N, Lyons J, Minter J (2004). MRI diagnosis of tears of the hip abductor tendons (gluteus medius and gluteus minimus). AJR Am J Roentgenol.

[16] Dienst M, Gödde S, Seil R, Hammer D, Kohn D (2001). Hip arthroscopy without traction: In vivo anatomy of the peripheral hip joint cavity. Arthroscopy.

[17] Dienst M, Seil R, Kohn DM (2005). Safe arthroscopic access to the central compartment of the hip. Arthroscopy.

[18] Docking SI, Cook J, Chen S, Scarvell J, Cormick W, Smith P, Fearon A (2019). Identification and differentiation of gluteus medius tendon pathology using ultrasound and magnetic resonance imaging. Musculoskelet Sci Pract.

[19] Domb BG (2010). Partial-thickness tears of the gluteus medius: rationale and technique for trans-tendinous endoscopic repair. Arthroscopy.

[20] Fearon AM, Scarvell JM, Cook JL, Smith PN (2010). Does ultrasound correlate with surgical or histologic findings in greater trochanteric pain syndrome? A pilot study. Clin Orthop Relat Res.

[21] Flack NAMS, Nicholson HD, Woodley SJ (2012). A review of the anatomy of the hip abductor muscles, gluteus medius, gluteus minimus, and tensor fascia lata. Clin Anat.

[22] Flack NAMS, Nicholson HD, Woodley SJ (2014). The anatomy of the hip abductor muscles. Clin Anat.

[23] Flato R, Passanante GJ, Skalski MR, Patel DB, White EA, Matcuk GRJ (2017). The iliotibial tract: imaging, anatomy, injuries, and other pathology. Skeletal Radiol.

[24] Ganderton C, Semciw A, Cook J, Moreira E, Pizzari T (2018). Gluteal loading versus sham exercises to improve pain and dysfunction in postmenopausal women with greater trochanteric pain syndrome: a randomized controlled trial. J Womens Health.

[25] Gottschalk F, Kourosh S, Leveau B (1989). The functional anatomy of tensor fasciae latae and gluteus medius and minimus. J Anat.

[26] Haliloglu N, Inceoglu D, Sahin G (2010). Assessment of peritrochanteric high T2 signal depending on the age and gender of the patients. Eur J Radiol.

[27] Hendry J, Biant LC, Breusch SJ (2012). Abductor mechanism tears in primary total hip arthroplasty. Arch Orthop Trauma Surg.

[28] Hernando MF, Cerezal L, Pérez-Carro L, Abascal F, Canga A (2015). Deep gluteal syndrome: anatomy, imaging, and management of sciatic nerve entrapments in the subgluteal space. Skeletal Radiol.

[29] Hyland S, Graefe S, Varacallo M (2020) Anatomy, Bony Pelvis and Lower Limb, Iliotibial Band (Tract). StatPearls In: StatPearls. Treasure Island (FL): StatPearls Publishing; 2020 Jan–. PMID: 30725782 30725782

[30] Ilizaliturri VMJ, Martinez-Escalante FA, Chaidez PA, Camacho-Galindo J (2006). Endoscopic iliotibial band release for external snapping hip syndrome. Arthroscopy.

[31] Kaltenborn A, Bourg CM, Gutzeit A, Kalberer F (2014). The Hip Lag Sign–prospective blinded trial of a new clinical sign to predict hip abductor damage. PLoS ONE.

[32] Khanduja V, Villar RN (2006). Arthroscopic surgery of the hip: current concepts and recent advances. J Bone Joint Surg Br.

[33] Kim CH, Lee SK, Kim JH, Yoon PW (2020). External snapping hip: classification based on magnetic resonance imaging features and clinical correlation. Hip Int.

[34] Kingzett-Taylor A, Tirman PF, Feller J, McGann W, Prieto V, Wischer T, Cameron JA, Cvitanic O, Genant HK (1999). Tendinosis and tears of gluteus medius and minimus muscles as a cause of hip pain: MR imaging findings. AJR Am J Roentgenol.

[35] Krishnamurthy G, Connolly BL, Narayanan U, Babyn PS (2007). Imaging findings in external snapping hip syndrome. Pediatr Radiol.

[36] Larsen E, Johansen J (1986). Snapping hip. Acta Orthop Scand.

[37] Lequesne M, Mathieu P, Vuillemin-Bodaghi V, Bard H, Djian P (2008). Gluteal tendinopathy in refractory greater trochanter pain syndrome: diagnostic value of two clinical tests. Arthritis Rheum.

[38] Lewis CL (2010). Extra-articular snapping hip: a literature review. Sports Health.

[39] Lindner D, Shohat N, Botser I, Agar G, Domb BG (2015). Clinical presentation and imaging results of patients with symptomatic gluteus medius tears. J Hip Preserv Surg.

[40] Long SS, Surrey DE, Nazarian LN (2013). Sonography of greater trochanteric pain syndrome and the rarity of primary bursitis. AJR Am J Roentgenol.

[41] Nakano N, Yip G, Khanduja V (2017). Current concepts in the diagnosis and management of extra-articular hip impingement syndromes. Int Orthop.

[42] Nolton EC, Ambegaonkar JP (2018). Recognizing and managing snapping hip syndrome in dancers. Med Probl Perform Art.

[43] Ortiz-Declet V, Chen AW, Maldonado DR, Yuen LC, Mu B, Domb BG (2019). Diagnostic accuracy of a new clinical test (resisted internal rotation) for detection of gluteus medius tears. J Hip Preserv Surg.

[44] Pelsser V, Cardinal E, Hobden R, Aubin B, Lafortune M (2001). Extraarticular snapping hip: sonographic findings. AJR Am J Roentgenol.

[45] Pfirrmann CW, Chung CB, Theumann NH, Trudell DJ, Resnick D (2001). Greater trochanter of the hip: attachment of the abductor mechanism and a complex of three bursae–MR imaging and MR bursography in cadavers and MR imaging in asymptomatic volunteers. Radiology.

[46] Plinsinga ML, Coombes BK, Mellor R, Vicenzino B (2020). Individuals with persistent greater trochanteric pain syndrome exhibit impaired pain modulation, as well as poorer physical and psychological health, compared with pain-free individuals: a cross-sectional study. Pain Med.

[47] Plinsinga ML, Ross MH, Coombes BK, Vicenzino B (2019). Physical findings differ between individuals with greater trochanteric pain syndrome and healthy controls: a systematic review with meta-analysis. Musculoskelet Sci Pract.

[48] Pozzi G, Lanza E, Parra CG, Merli I, Sconfienza LM, Zerbi A (2017). Incidence of greater trochanteric pain syndrome in patients suspected for femoroacetabular impingement evaluated using magnetic resonance arthrography of the hip. Radiol Med (Torino).

[49] Reimer LCU, Jacobsen JS, Mechlenburg I (2019). Hypermobility among patients with greater trochanteric pain syndrome. Dan Med J.

[50] Robertson WJ, Gardner MJ, Barker JU, Boraiah S, Lorich DG, Kelly BT (2008). Anatomy and dimensions of the gluteus medius tendon insertion. Arthroscopy.

[51] Segal NA, Felson DT, Torner JC, Zhu Y, Curtis JR, Niu J, Nevitt MC (2007). Greater trochanteric pain syndrome: epidemiology and associated factors. Arch Phys Med Rehabil.

[52] Sutter R, Kalberer F, Binkert CA, Graf N, Pfirrmann CWA, Gutzeit A (2013). Abductor tendon tears are associated with hypertrophy of the tensor fasciae latae muscle. Skeletal Radiol.

[53] Tortolani PJ, Carbone JJ, Quartararo LG (2002). Greater trochanteric pain syndrome in patients referred to orthopedic spine specialists. Spine.

[54] Tsutsumi M, Nimura A, Akita K (2019). The gluteus medius tendon and its insertion sites: an anatomical study with possible implications for gluteus medius tears. J Bone Joint Surg Am.

[55] Walker-Santiago R, Ortiz-Declet V, Maldonado DR, Wojnowski NM, Domb BG (2019). The modified resisted internal rotation test for detection of gluteal tendon tears. Arthrosc Tech.

[56] Westacott DJ, Minns JI, Foguet P (2011). The diagnostic accuracy of magnetic resonance imaging and ultrasonography in gluteal tendon tears–a systematic review. Hip Int.

[57] Williams BS, Cohen SP (2009). Greater trochanteric pain syndrome: a review of anatomy, diagnosis and treatment. Anesth Analg.

